# Dietary pattern analysis: a comparison between matched vegetarian and omnivorous subjects

**DOI:** 10.1186/1475-2891-12-82

**Published:** 2013-06-13

**Authors:** Peter Clarys, Peter Deriemaeker, Inge Huybrechts, Marcel Hebbelinck, Patrick Mullie

**Affiliations:** 1 Faculty of Physical Education and Physiotherapy, Department of Human Biometrics and Biomechanics, Vrije Universiteit Brussel, Brussels, Belgium; 2 Erasmus University College, Brussels, Belgium; 3 International Agency for Research on Cancer (IARC), Dietary Exposure Assessment Group (DEX), Lyon, France; 4 International Prevention Research Institute (iPRI), Ecully (Lyon), France

## Abstract

**Background:**

Dietary pattern analysis, based on the concept that foods eaten together are as important as a reductive methodology characterized by a single food or nutrient analysis, has emerged as an alternative approach to study the relation between nutrition and disease. The aim of the present study was to compare nutritional intake and the results of dietary pattern analysis in properly matched vegetarian and omnivorous subjects.

**Methods:**

Vegetarians (n = 69) were recruited via purposeful sampling and matched non-vegetarians (n = 69) with same age, gender, health and lifestyle characteristics were searched for via convenience sampling. Two dietary pattern analysis methods, the Healthy Eating Index-2010 (HEI-2010) and the Mediterranean Diet Score (MDS) were calculated and analysed in function of the nutrient intake.

**Results:**

Mean total energy intake was comparable between vegetarians and omnivorous subjects (p > 0.05). Macronutrient analysis revealed significant differences between the mean values for vegetarians and omnivorous subjects (absolute and relative protein and total fat intake were significantly lower in vegetarians, while carbohydrate and fibre intakes were significantly higher in vegetarians than in omnivorous subjects). The HEI and MDS were significantly higher for the vegetarians (HEI = 53.8.1 ± 11.2; MDS = 4.3 ± 1.3) compared to the omnivorous subjects (HEI = 46.4 ± 15.3; MDS = 3.8 ± 1.4).

**Conclusions:**

Our results indicate a more nutrient dense pattern, closer to the current dietary recommendations for the vegetarians compared to the omnivorous subjects. Both indexing systems were able to discriminate between the vegetarians and the non-vegetarians with higher scores for the vegetarian subjects.

## Background

Dietary pattern analysis, based on the concept that foods eaten together are as important as a reductive methodology characterized by a single food or nutrient analysis, has emerged as an alternative approach to study the relation between nutrition and disease [1-4]. As reviewed by Hu [4], dietary pattern analysis is a different method to examine the effect of overall diet: food and nutrients are not eaten in isolation, and the ‘single food or nutrient’ approach will not take into account the complex interactions between foods and nutrients. Two major methods are used to reduce complex dietary data: a hypothesis-oriented approach using previous information to stratify a dietary pattern and a statistical approach using study-specific data to rank individuals (principal component analysis or reduced rank regression models) [5,6]. The Healthy Eating Index (HEI) and the Mediterranean Diet Score (MDS) are two frequently used hypothesis-oriented approaches [7-10]. The HEI represents the degree to which a dietary pattern conforms to official guidelines summarized in the United States Department of Agriculture Food Guide Pyramid [11-13]. This system was first proposed in 1995 by Kennedy et al. and further refined in function of evolving Dietary Guidelines and intakes in function of recommended levels, resulting in a 2005 and 2010 version (abbreviated in this paper respectively as HEI-1995, HEI-2005, and HEI-2010). In contrast to the original version the more recent versions of the HEI use an energy-adjusted density approach, limiting the possible confounding effect of total energy intake [12,13]. The most recent version, the HEI-2010, is made up of 12 components, 9 adequacy components (total fruit, whole fruit, total vegetables, greens and beans, whole grains, dairy, total protein foods, seafood and plant proteins, fatty acids) and 3 moderation components (refined grains, sodium, empty calories) [13]. The MDS is composed of seven desirable components (cereals, vegetables, fruits and nuts, legumes, fish, a high dietary ratio of mono-saturated to saturated fatty acids and moderate alcohol consumption) and two undesirable components (meat and dairy food products). Both systems use adequacy or desirable and moderation or undesirable scoring systems but for different components.

Vegetarians differ from omnivorous subjects in their eating pattern by the exclusion of meat and fish (lacto-ovo-vegetarians) or by the exclusion of all animal derived products (strict vegetarians or vegans). On the other hand, these restricted diets are generally higher in vegetables, fruits, and whole grains. Nutritional analysis indicates lower protein, (saturated) fat and higher carbohydrate and fibre intake. In most of the studies, vegetarian macro- and micronutrient intake is closer to the recommendations [14-17].

Higher scores on HEI-2005 and MDS are related with positive health outcomes [18,19] whilst several health advantages are attributed to the vegetarian diet [20,21]. Since both indexing systems vary in the definition of optimal diet quality and in their scoring mechanism they may differ in their sensitivity to capture differences between vegetarian and omnivorous subjects. Indeed, the more recent versions of the HEI (HEI-2005 and HEI-2010) do not require any single commodity in order to achieve a high score whilst the MDS includes specific sources. Especially the possibility to include both animal and plant protein sources in the HEI-2005 and HEI-2010 make these indexes amenable for vegetarian diets. In contrast, the MDS uses more traditional components such as meat and fish, which may be a weakness when analyzing alternative diets [12,13,18].

Up to now, the number of studies using these indices for the comparison between vegetarians and non-vegetarians is limited [12,22]. Kennedy et al. [22] calculated the HEI-1995 for 642 vegetarians compared to 9372 omnivorous subjects from the American Continuing Survey of Food Intake by Individuals (CFSSI) between 1994 and 1996. The HEI-1995 was significantly lower for the vegetarians compared to the omnivorous subjects. In contrast, calculating the HEI-2005 on data of the National Health and Nutrition Examination Survey 1999–2004, Farmer and colleagues found that the dietary quality of non-dieting vegetarians was superior compared to their omnivorous counterparts. However, the profound adaptations between the original and the latter iterations of the HEI, make a comparison over the different versions difficult.

In the study of Costacou et al. [23], principal component analysis revealed four dietary patterns amongst 28.034 Greek participants of the EPIC study: a Mediterranean, a Vegetarian, a Sweet and a Western dietary pattern. In that study, the data driven Mediterranean-resembling component was strongly positively associated with the hypothesis driven MDS whilst the Vegetarian approximating component was unrelated with the MDS. The latter indicates that amongst these Greek participants, subjects with a vegetarian-type diet consume food groups not matching properly with the components of the MDS. This points again to the possible limitations of the MDS for the evaluation of restricted diets.

The literature on HEI scores for vegetarians remains equivocal and to the best of our knowledge no comparison between vegetarian and omnivorous subjects has been made using the MDS.

In most studies, self-selected or post examination detected vegetarians are compared with standard population references or a non-matched sample. In order to reduce lifestyle bias [24], comparison according to the matched samples principle is advised [17,25]. It was the aim of this study to compare the nutrient intakes and the diet quality in properly matched vegetarian and omnivorous subjects. Two dietary pattern analysis methods, the HEI-2010 and MDS were calculated and analysed in function of the followed diet.

## Methods

Since no register of vegetarian subjects exists, purposeful sampling was conducted. Vegetarian subjects were actively recruited in the Flemish region of Belgium through advertising in health food shops, and on the website of vegetarian associations. Inclusion criteria were being a vegetarian for at least one year and being at least 18 years of age. Vegetarianism was defined as abstinence of meat, game, poultry, and fish in the diet. This purposeful sampling resulted in the collaboration of 69 vegetarians. Matching was done either by the vegetarian subjects recruiting themselves a proxy of the same age, gender, health and lifestyle characteristics (n = 20 vegetarians) or by matching according to sex, age, BMI, level of physical activity, tobacco use and alcohol consumption using a data set of 1520 omnivorous subjects [14,15]. All subjects completed a 3 days food diary (2 week days and 1 weekend day).

All diaries contained an instruction manual including portion size or weight or volume of common household measures. Volunteers were asked to weigh the consumed food items when possible or otherwise to indicate portion size of the common food items. Analysis of the food records was performed with the BECEL software program, using Belgian nutrient composition tables. The HEI-2010 and the MDS were computed as described earlier [13,18]. For both indexes population ratio scores over the 3 days were calculated. The possible scores for HEI-2010 ranged from 0 to 100 and for MDS from 0 to 9, with a higher score for a healthier pattern.

For baseline characteristics mean and standard deviation were calculated. To improve clarity, the components of HEI and MDS were expressed as mean and standard deviation. Due to the general non-normality of the data distribution after visual control and Kolmogorov-Smirnov test, differences between vegetarians and omnivorous subjects were tested with the Mann–Whitney *U* test. Ethical clearance was obtained by the Ethical Committee of the Vrije Universiteit Brussel.

## Results

More vegetarian women (n = 49) than men (n = 20) were prepared to participate in the study and completed the 3-days diary. Mean age of the vegetarian women and men was respectively 35 ± 12 and 39 ± 14 years, for the omnivorous subjects mean age was respectively 36 ± 12 and 40 ± 14 years (both p >0.05). Mean BMI was 22 ± 4 kg/m^2^ for the vegetarian women and 23 ± 3 kg/m^2^ for the vegetarian men compared to respectively 23 ± 4 and 24 ± 3 kg/m^2^ for their omnivorous counterparts; both p > 0.05. Comparison of age, BMI, physical activity, smoking habits and physical activity indicated a proper matching of all the subjects [15].

Mean total energy intake was comparable between vegetarians and omnivorous subjects (p > 0.05), (Table 1). Macronutrient analysis reveals significant differences between the mean values of the vegetarians and the omnivorous subjects. Absolute and relative protein intake was significantly lower for the vegetarians compared to the omnivorous subjects. Absolute and relative total fat intake was significantly lower for the vegetarians compared to the omnivorous subjects. Saturated, mono-unsaturated and cholesterol intakes were significantly lower for the vegetarians compared to the omnivorous subjects. Both, absolute and relative carbohydrate intake were significantly higher for the vegetarians compared to the omnivorous subjects. Fibre intake was almost double for the vegetarians compared to the omnivorous subjects.

**Table 1:** Energy and macronutrient intake of the study participants (mean and SD)

**Variable**	**Vegetarians (n = 69)**	**Omnivores (n = 69)**	P-value*
Energy (kcal)	2070 (570)	2120 (585)	0.612
Proteins (g)	66.4 (18.4)	79.3 (23.5)	<0.001
Fat (g)	68.1 (22.5)	81.0 (29.8)	0.005
Saturated fatty acids (g)	22.2 (10.0)	30.2 (12.3)	<0.001
Mono-unsaturated fatty acids (g)	20.8 (9.2)	31.0 (13.2)	<0.001
Poly-unsaturated fatty acids (g)	14.2 (6.7)	13.7 (6.8)	0.707
Cholesterol (mg)	116.0 (84.1)	213.5 (97.3)	<0.001
Carbohydrates (g)	286.6 (92.0)	254.9 (75.6)	0.029
Fibres (g)	29.0 (12.5)	16.6 (5.3)	<0.001
Alcohol (g)	0.9 (1.6)	6.8 (17.8)	<0.001
Proteins (energy-percent)	13.0 (2.2)	15.2 (3.1)	<0.001
Fat (energy-percent)	29.8 (6.6)	33.8 (7.6)	0.001
Saturated fatty acids (energy-percent)	9.7 (3.2)	12.6 (3.4)	<0.001
Mono-unsaturated fatty acids (energy-percent)	9.1 (3.3)	12.9 (4.1)	<0.001
Poly-unsaturated fatty acids (energy-percent)	6.3 (2.8)	5.7 (2.1)	0.163
Carbohydrates (energy-percent)	55.2 (7.3)	48.4 (7.4)	<0.001

* Mann–Whitney *U* test.

The HEI-2010 was significantly higher for the vegetarians (53.8 ± 11.2) compared to the omnivorous subjects (46.4 ± 15.3), (p = 0.001), (Table 2). More detailed analysis of the different components of the HEI indicated a more favourable score for 8 of the 12 components for the vegetarians compared to the omnivorous subjects. A significant (p < 0.05) higher score was found for total fruit, whole fruit and, seafood and plant proteins. Total vegetables, whole grains, and empty calories showed a trend (p < 0.1) for a higher score for the vegetarians compared to the omnivorous subjects. The components total protein food, fatty acids and refined grains where significantly higher (p <0.05) for the omnivorous subjects compared to the vegetarians.

**Table 2:** Total and component Healthy Eating Index-2010 scores for vegetarians and omnivores (mean and SD)

	**Vegetarians (n = 69)**	**Omnivores (n = 69)**	P value*
Total fruit (0–5 points)	3.8 (1.5)	2.6 (1.8)	<0.001
Whole fruit (0–5 points)	3.9 (1.5)	3.1 (1.9)	0.005
Total vegetables (0–5 points)	2.1 (1.4)	1.7 (1.3)	0.078
Greens and beans (0–5 points)	0.9 (1.6)	1.0 (1.7)	0.780
Whole grains (0–10 points)	9.1 (2.2)	8.2 (3.2)	0.052
Dairy (0–10 points)	3.5 (3.1)	2.8 (2.5)	0.153
Total protein foods (0–5 points)	3.0 (1.9)	4.7 (0.7)	<0.001
Seafood and plant proteins (0–5 points)	1.7 (1.6)	0.9 (1.0)	0.001
Fatty acids (0–10 points)	2.6 (2.2)	3.9 (4.0)	0.012
Refined grains (0–10 points)	3.3 (0.4)	4.2 (0.5)	<0.001
Sodium (0–10 points)	7.9 (2.9)	7.4 (3.2)	0.329
Empty calories (0–20 points)	7.3 (8.4)	5.0 (7.8)	0.099
**Total score (0–100 points)**	**53.8 (11.2)**	**46.4 (15.3)**	**0.001**

* Mann–Whitney *U* test.

The obtained MDS was higher for the vegetarians (4.3 ± 1.3) compared to the omnivorous subjects (3.8 ± 1.4), (p = 0.040), (Table 3). Vegetarians had a higher number of subjects above the median for 3 (vegetables, fruit and nuts, legumes) out of the 6 desirable components, 1 (meat) out of the 2 undesirable components, and the moderation component (alcohol). The mean values of the desirable MDS components indicate significant lower intakes of cereals and fish and significantly higher intakes of fruits and nuts for the vegetarian compared to the omnivorous subjects. For legumes a trend towards a higher intake (p < 0.1) was detected when comparing vegetarian with omnivorous subjects. Dairy intake, an undesirable component, was significantly higher for the vegetarian compared to the omnivorous subjects. The intake of alcohol, the moderation component of the MDS, was significantly lower for the vegetarians compared to the omnivorous subjects.

**Table 3:** Mediterranean Diet Score (MDS) and intakes for the different components (mean and SD) for vegetarians and omnivores

	**MDS**		**Mean intake**		
	**Vegetarians**	**Omnivores**	**Vegetarians**	**Omnivores**	
**Component**	**n(%)**	**n(%)**	**Mean ± SD**	**Mean ± SD**	P*
Cereals	29 (42)	40 (58)	292.5 ± 138.2 g	354.6 ± 197.6 g	0.034
Vegetables	40 (58)	29 (42)	207.5 ± 151.0 g	190.7 ± 162.5 g	0.531
Fruits and nuts	41 (59)	28 (41)	264.3 ± 227.8 g	163.8 ± 159.1 g	0.003
Legumes	19 (28)	12 (17)	12.8 ± 31.6 g	5.4 ± 15.3 g	0.082
Dairy	26 (38)	43 (62)	256.1 ± 182.3 g	165.0 ± 161.2 g	<0.002
Fish	0 (0)	39 (57)	0.0	38.6 ± 55.3 g	<0.001
MUFA/SFA	34 (49)	35 (51)	1.03 ± 0.47	0.99 ± 0.31	0.538
Meat	69 (100)	0 (0)	0.0	158.8 ± 68.4 g	<0.001
Alcohol	39 (57)	30 (43)	0.9 ± 1.6 g	6.8 ± 17.8 g	0.007
**Total MDS**	**4.3 ±1.3**	**3.8 ±1.4**			0.040

n (%) = number and percentage of subjects reaching the median; * Mann–Whitney *U* test for intake and Total MDS comparison between vegetarians and omnivores.

## Discussion

In the present study nutrient intake and dietary indices, HEI-2010 and MDS, were calculated for properly matched vegetarian and omnivorous subjects.

Nutrient analysis indicates significant differences between the vegetarians and the omnivorous subjects. In general macro- and micronutrient intake was closer to the recommendation for the vegetarians compared to the non-vegetarians. These data are completely in agreement with the results of Farmer et al. [12] and Kennedy et al. [22] reporting a more nutrient dense intake for the vegetarians compared to the non-vegetarians. Similar nutrient intakes are reported in most of the studies on vegetarians [21].

The recently released HEI-2010 allowed the discrimination between a vegetarian and omnivorous dietary pattern of properly matched health conscious subjects with a higher overall quality index for the vegetarians compared to the omnivorous subjects.

This is completely in line with the results of Farmer et al. [12] using the HEI-2005 where non-dieting vegetarians obtained a higher score compared to non-dieting omnivores. In contrast, using the 10-component HEI-1995 method, Kennedy et al. [22], reported lower quality scores for the vegetarians compared to the omnivorous subjects. The results of Farmer et al. [12] together with ours confirm the applicability of the more recent proposed HEI methods - with refinement towards vegetables and plant protein components - to estimate the quality of more restricted plant based diets.

The MDS resulted equally in higher values when applied on the vegetarians compared to the omnivorous subjects, reaching only borderline significance. The latter may be inherent to the method of this model where scores are attributed based on the median value of the different components. Although most of the plant-based MDS components seem to be higher in the vegetarian compared to the omnivorous subjects, they do not always reach significance. In the case of legumes, this may be due to the considerable intake variability and the small sample size.

In this model vegetarians can never obtain scores for the desirable component fish whilst omnivorous subjects always score above the median for the undesirable component meat. Hence, the MDS loses discriminative power on 2 of the 9 components. This may be an indication that the MDS needs further elaboration with regard to more plant-based nutrition with a more balanced or possible contribution for alternative components [26].

Indeed, Costacou et al. [23], did not find a relation between the MDS and the “Vegetarian like component” but identified clusters of dietary patterns, sharing similar dietary behavior and showing a strong preference to the “Mediterranean” and the closely related “Vegetarian like component”. The discriminative power between two populations with a rather healthy eating pattern seems to be weaker for the MDS compared to the HEI-2010. It was indeed demonstrated that the studied population (vegetarians and controls) showed healthier eating and lifestyle habits compared to the Belgian population [27]. Nevertheless, for both methods scores for vegetarians and omnivores were only weak to moderate. This may be an indication that the nutritional intake of both groups needs further improvement. The lower scores for the MDS indicate equally that this model does not allow scoring of components, which may be part of a healthy diet besides the components of the Mediterranean Diet. The more recent released versions of the HEI (HEI-2005 and HEI 2010) allow several types of healthy diets.

On the other hand, the combination of components from the different systems may result in conflicting information. In the HEI-2010 the dairy component is an “adequacy” component whilst it is an “undesirable” component in the MDS. In agreement with the Farmer study we found a higher dairy intake for the vegetarians compared to the non-vegetarians [12]. Indeed, the dairy component may be an important calcium source but may equally have its impact on the fatty acid balance. The latter, in combination with the absence of fish in the diet, may be an explanation for the weak score on the fatty acid ratios in the HEI-2010 and MDS for the vegetarians.

A weakness of the present study is the purposeful sampling of vegetarian subjects and the matching based -amongst other- on health related parameters (BMI, physical activity level, smoking habits). The latter resulted in a health conscious sample of omnivores which was not representative for the Flemish population [15,27]. Also, none of the used indexes was specifically developed for a Belgian population. The HEI is a measure used to asses diet quality in terms of conformance to the Dietary Guidelines for Americans [13] whilst the MDS is based on a more Southern European region consumed pattern [23,26]. However, the latest Belgian food Consumption survey indicated substantial differences between the usual food consumption and the Belgian food based dietary guidelines [26]. The reported inadequacies are comparable with those reported for the American population (high protein and fat intake, low fruit and vegetable intake, high saturated fatty acid and added sugar intake). Moreover, the Active Food Triangle as used in Belgium for the promotion of a balanced diet shows great resemblance with the USDA Food Pyramid [28]. In the most recent update of the HEI [13] it was equally assumed that the HEI-2010 could be considered valid for different ethnic and cultural groups.

Indeed, several reports indicate that the older versions of the HEI as well as the MDS used in different European populations add valuable information to the estimation of diet quality [23,29]. The discriminative power for two small health conscious groups may be considered an indication for the robustness of the HEI-2010 with sufficient sensitivity for omnivorous and more restricted diets. Future research should not only use these indices on larger, representative samples but use in longitudinal designs may help to unravel the relation between diet quality and different health outcomes.

## Conclusions

In conclusion, the vegetarian diet as consumed in our sample is closer to the nutritional recommendations compared to the omnivorous diet. The latter resulted in a higher HEI-2010 score and MDS for the vegetarians compared to the matched omnivorous subjects. The updated HEI (HEI-2010) method allows adequate scoring of restricted diets. Despite two components not allowing alternatives when comparing vegetarians with non-vegetarians (meat and fish), the MDS is still able to discriminate between the vegetarian and omnivorous subjects.

## Competing interests

The authors declare that they have no competing interests.

## Authors’ contributions

PC conceived the original idea of the publication, performed the study and wrote the first draft; PM analysed the data, PM, PD, IH, MH critically appraised the paper. All authors read and approved the final manuscript.
